# Research on Anxiety of Learning Chinese as a Second or Foreign Language in and Outside Mainland China: A Systematic Review of the Literature 1999–2020

**DOI:** 10.3389/fpsyg.2022.843858

**Published:** 2022-03-10

**Authors:** Shuangyun Yao, Dujuan Zhang, Qian Shen

**Affiliations:** ^1^Research Center for Language and Language Education, Central China Normal University, Wuhan, China; ^2^School of Foreign Studies, Zhongnan University of Economics and Law, Wuhan, China

**Keywords:** learning anxiety, Chinese as a second or foreign language, mainland Chinese journals, international journals, bibliometric analysis

## Abstract

This paper reviews research on anxiety of learning Chinese as a second or foreign language (CSL/CFL) in and outside mainland China. This review involves 52 Chinese language articles identified in leading journals from the Chinese National Knowledge Infrastructure (CNKI) database (中国知网) and 42 English language articles from the Web of Science and ERIC database published during the period of 1999 to 2020. By adopting bibliometric analysis and content analysis, this study compares the topical issues and methodological approaches of research on CSL/CFL learning anxiety published in leading Chinese and international journals. The review found that, compared with mainland Chinese scholars, international researchers examined a broader range of topical issues from multidimensional perspectives. While most Chinese empirical studies are dominated by the quantitative approach, qualitative methods such as classroom observations and in-depth interviews were also widely adopted by international researchers. The analysis also revealed that although Chinese scholars had drawn on well-established theories and concepts originating from foreign language anxiety (FLA) research, their role in CSL/CFL anxiety research is limited and peripheral. Consequently, we conclude this review with recommendations that encourage mainland Chinese researchers to be well informed by the updated theoretical perspective and methodological approaches such as the utilization of social network analysis and the integration of Information Communications Technology in language education.

## Introduction

A great variety of emotions experienced by language learners have attracted scholarly attention from various linguistic, educational, and sociocultural contexts (Jain and Sidhu, 2013; Dewaele, 2018; Miyahara, 2019). Among them, a situation-specific anxiety, that is, Foreign Language Anxiety (FLA) proposed by Horwitz et al. (1986), is responsible for students’ negative affective reactions to foreign language learning. This complicated psychological phenomenon related to foreign language learning is defined as “a distinct complex of self-perceptions, beliefs, feelings, and behaviors related to classroom language learning arising from the uniqueness of the language learning process” (Horwitz et al., 1986, p. 128). In order to accurately measure learners’ anxiety of learning a foreign language, a standard instrument, the Foreign Language Classroom Anxiety Scale (FLCAS), was designed and developed, and which has been widely adopted in a range of empirical studies (e.g., Saito et al., 1999; Kitano, 2001; Park, 2014; Weng, 2020). These studies on FLA are conducted in different language education contexts, such as Japanese (Aida, 1994; Djafri and Wimbarti, 2018), Korean (Kim, 2000; Lee, 2021), Spanish (Noels, 2001; Cordeiro, 2019), French (Rodríguez and Abreu, 2003; Bosmans and Hurd, 2016), and Chinese (Le, 2004; Zhao, 2013) providing cross-linguistic and cross-cultural evidence for the pervasive existence of anxiety in foreign language learning. Since Chinese is distinctly different from alphabetic languages such as English due to its unique tone system and logographic writing system (Shen and Xu, 2015, p. 82), it posts enormous challenge for Chinese as a second or foreign language (CSL/CFL) learners, especially native-English learners, which reflects partly on the high drop-out rate of CSL/CFL classes (Luo, 2011). Previous studies have revealed that the withdrawal rate is remarkably higher among learners with higher levels of FLA (Bailey et al., 2003; Xiao and Wong, 2014). Therefore, there is plausible reason to believe that anxiety is a highly relevant issue among CSL/CFL learners. However, in contrast to the abundant research on English language anxiety (ELA) with a long history, research on anxiety of learning CSL/CFL is not only scarce but only a recent concern. During the preceding two decades, most studies involving CSL/CFL education have focused on language pedagogy (e.g., Mao, 2010; Tao, 2012), Chinese character, phonetic, lexical, and grammatical learning (e.g., Wu et al., 2006; Wen, 2010; Fan, 2013; Luan, 2013), language testing (Wang, 2008), and teacher development (Guo, 2012), while affective factors such as attitudes, motivation, beliefs, and anxiety have not received adequate attention (Yu, 2010). In particular, language researchers started to steer their attention to the anxiety level of CSL/CFL learners in the late-1990s (Qian, 1999; Jiang and Ramsay, 2005). Since then, an increasing number of studies on anxiety of CSL/CFL learners have been published in and outside mainland China. Therefore, to depict a whole picture of the current literature on anxiety level specific to CSL/CFL learners, a systematic review on this issue is urgently needed.

However, we notice that the academic exchanges and communication between scholars who publish papers in mainland China and those who publish in international journals^1^ are insufficient (Gong et al., 2018). One piece of evidence is that few articles published in mainland China concerning CSL/CFL learners’ anxiety were cited in related articles published in international journals. It seems that the two groups of researchers may not be fully appreciating what their counterparts have achieved in this field due to inadequate bilingual competence and differences in academic practices (Gong et al., 2018, 2020a,c). Moreover, although two reviews of studies on anxiety of learning CSL/CFL were retrieved (e.g., Chen, 2018; Xu, 2018), they were published in non-CSSCI journals in mainland China with limited impact. Despite the fact that the existing two reviews did provide some insights into this field, they only addressed the studies published in mainland China with a limited time span. Therefore, it is a pressing need to provide a systematic review of research on anxiety of CSL/CFL learners conducted both in and outside mainland China to reflect the topic issues and methodological approaches so that informative mutual exchanges can be facilitated. Moreover, given that the number of students learning CSL/CFL outside mainland China is significant and is still steadily increasing (Ministry of Education of the People’s Republic of China, 2019) as well as the anxiety of learning CSL/CFL being prevalent among non-Chinese learners abroad (Luo, 2011), it was presumed that mainland Chinese researchers working on anxiety of CSL/CFL learners could learn much from relevant studies published in international journals. To verify this assumption, it is vital to find out to what extend have mainland Chinese researchers referred to studies on anxiety of learning CSL/CFL conducted in the international context. To achieve these ends, this review attempts to address the following two research questions:

RQ1: What topical issues and methodological approaches can be identified in studies on anxiety of learning CSL/CFL published in mainland Chinese journals and international journals?

RQ2: How have mainland Chinese scholars drawn on research on the anxiety of learning CSL/CFL conducted outside mainland China?

## Methodology

### Database Selection

In view of the sociocultural and historical differences between diverse learning contexts in the greater China region, this review was confined to journals published in mainland China, excluding publications from Chinese Taiwan, Hong Kong SAR, and Macau SAR (Gong et al., 2018). Moreover, because of our concern for both comprehensive coverage and potential impact of the relevant literature in this scope, we selected three databases, that is, the China National Knowledge Infrastructure (CNKI, 中 国 知 网), the Web of Science (WoS), and the Education Resources Information Center (ERIC). CNKI was chosen due to the fact that it is the largest academic journal database in mainland China, covering comprehensive integrated knowledge resources of all disciplines. WoS was selected as another primary database because it offers subscription-based access to many different databases that provide comprehensive citation data for multiple academic disciplines (Steinhardt et al., 2017). In addition, we also chose ERIC, a comprehensive full-text database specialized in educational research, as a complement to the two preceding databases. Generally, these three databases provide researchers with a wealth of data to understand the latest progress and trending research issues of diverse disciplines (Kuang et al., 2016).

### Article Selection

After preliminary searching string-based selection, we found that the first Chinese language publication concerning anxiety of learning CSL/CFL dates back to 1999 (cf., Qian, 1999) while the first English language article in this field was published in 2005 (cf., Jiang and Ramsay, 2005). Also, as we could not guarantee that all related papers published in 2021 could be collected, we limited this review to research published during the years 1999–2020 to ensure the accuracy and validity of the findings. Our first step was to use search strings to search and select articles regarding anxiety in Chinese language learning published in mainland China. Specifically, the search strings [topics = (汉语“Chinese language” + 留学生“overseas students” + 来华“study abroad in China”) *焦虑“anxiety”] were adopted in the CNKI database, which resulted in 401 articles being found. Secondly, to identify relevant English language publications, the search strings [topic = (Chinese language AND anxiety)] were used in WoS and ERIC, which yielded 421 and 116 articles respectively. Next, these two collections of articles from the two databases, i.e., WoS and ERIC, were compared in case of data duplication and 16 articles were found to be overlapping and consequently excluded from one of the collections.

**FIGURE 1:**
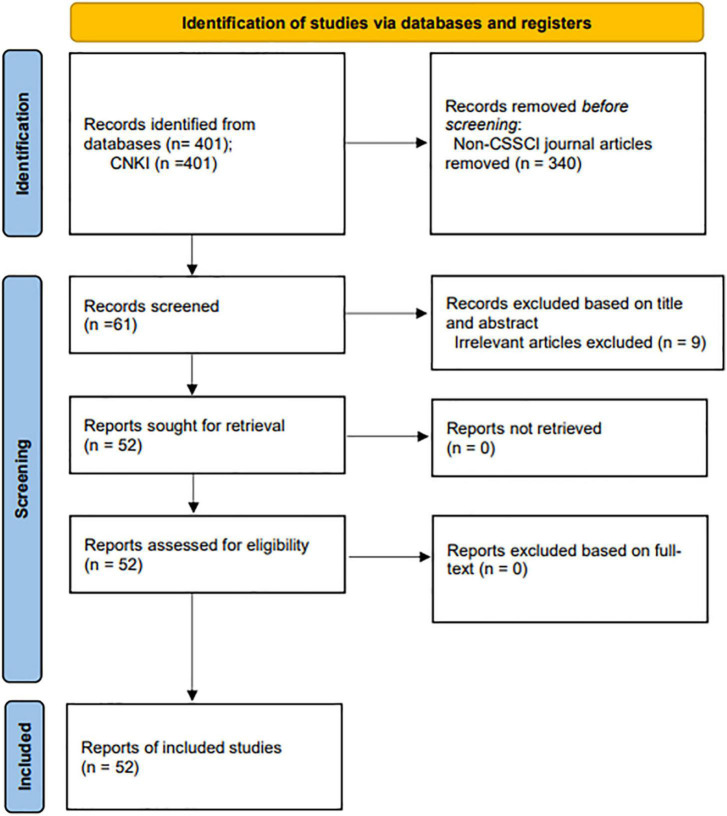
Chinese language articles’ selection process.

**FIGURE 2:**
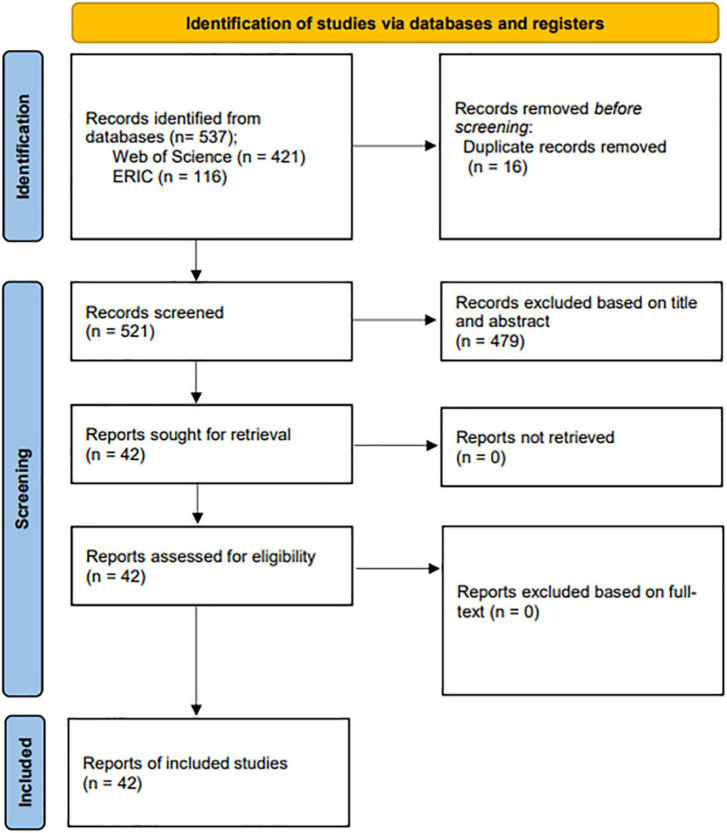
English language articles’ selection process.

As a result, a total of 401 Chinese publications from CNKI database and 521 English publications from WoS database and ERIC database were found. Moreover, considering the quality and potential impact of the relevant studies, we further limited our review to core journals listed in the China Social Sciences Citation Index (CSSCI): an interdisciplinary citation database that covers “the top journals with high academic quality from thousands of journals in China” (Ge, 2019, p. 48). Hence, a total of 61 articles were found. To further identify the relevant studies related to anxiety of CSL/CFL learners, the titles and abstracts of the resulting 582 articles were then examined and evaluated by all three members of the team. After the thorough evaluation, 94 articles were identified as relevant, including 52 Chinese language articles (see Figure 1) published in mainland China and 42 English language articles (see Figure 2) published in international journals. The PRISMA flow diagrams presented in Figures 1, 2 follow the guidance from PRISMA statement (Page et al., 2021).

### Analysis and Tools

To identify the topical issues of research on anxiety of learning CSL/CFL, we conducted the bibliometric analysis and content analysis. To be specific, this review inspected the bibliometric indicators of the selected publications, which included the publication year and keyword. Keyword co-occurrence analysis as well as keyword frequency analysis were carried out, aiming at detecting the primary content and emerging trends of a body of scientific knowledge (Callon et al., 1983; Chen, 2004, 2006). Keyword co-occurrence means the common occurrence and sharing of the same keywords across different papers, revealing core research domains and cognitive structures (Su and Lee, 2010). The embedded bibliometric analysis tool in the CNKI database and the WoS database, as well as Vosviewer, a software that can build and visualize bibliometric networks structure, were used to detect and visualize the findings (Van Eck and Waltman, 2010). Meanwhile, content analysis was adopted to examine the topics addressed in each of these selected articles. To be precise, the titles, abstracts, and conclusions of the reviewed publications were studied by three members of the team to determine the addressed topical issues. Whenever necessary, we also read the full text to ascertain their topics. This categorization of the topical issues was based on an existing classification of themes in FLA research (e.g., Luo, 2013), which provided us basic criteria for theme classification in CSL/CFL anxiety research. During the review process, new themes were appended or subcategories within each existing theme were refined and revised to capture what was revealed. Through the categorization process conducted by two members individually, we have identified four categories, namely, measure of CSL/CFL anxiety, factors associated with CSL/CFL anxiety, effects of CSL/CFL anxiety, and classroom practices to reduce CSL/CFL anxiety. As “the most widely used measure of interjudge reliability” (Perreault and Leigh, 1989, p. 137), Cohen’s (1960) kappa was used to assess the intercoder reliability for each categorized articles. And kappa here was 0.871, indicating a high level of reliability. The content analysis enables us to precisely identify the topic as well as the detailed relevant content of individual article for in-depth analysis while the bibliometric analysis visualize a holistic network map of the topical issues in mainland Chinese and international journals for global comparison. To identify the methodological approaches on anxiety of learning CSL/CFL, methodological sections or relevant descriptions were examined to identify which methodological paradigm had been adopted.

To address the second research question, the citation lists of the 52 selected Chinese language articles were analyzed to understand how these studies had drawn upon previous research on the anxiety of learning CSL/CFL published outside mainland China. Citations have been interpreted as a reference to some previously published source of information that is relevant to the argument the author wants to make (Baird and Oppenheim, 1994). They are seen as an index of how an article builds on a previous body of research. Therefore, an analysis of the type and number of citations can help to shed light on the second research question. In total, 318 citations were identified from the 52 selected publications. In an attempt to detect how these Chinese language articles had drawn upon their English counterparts, two members of our team first classified all the citations into Chinese language publications and English language publications. And while examining the topical issues of these citations through reading their titles and abstracts, we noted that a portion of articles in both Chinese and English addressing issues of second language acquisition (SLA) are not associated with FLA. Thus they were classified as separate groups. Consequently, the 318 citations were categorized into eight types: (1) Chinese language publications on CSL/CFL anxiety research; (2) Chinese language publications on English or other language anxiety research; (3) Chinese language publications on SLA^2^; (4) Chinese language publications unrelated to FLA or SLA; (5) English language publications on CSL/CFL anxiety research; (6) English language publications on English or other language anxiety research; (7) English language publications on SLA; and (8) English language publications unrelated to FLA or SLA. Two members of our team conducted the citation analysis based on these eight categories individually. And to measure the intercode agreement, Cohen’s (1960) kappa was also used to assess the intercoder reliability for each category. And kappa here was 0.846, showing a high level of reliability.

## Results

### Topical Issues

#### General Observations on Topical Distributions of the Studies

The thematic analysis indicated that the 94 selected articles (52 Chinese language articles and 42 English language articles) could be categorized into four groups: measure of CSL/CFL anxiety, factors associated with CSL/CFL anxiety, effects of CSL/CFL anxiety, and classroom practices to reduce CSL/CFL anxiety. The number of articles and its corresponding proportion within each category is specified in Table 1. To further detect whether there is any difference between the topical concerns in mainland Chinese articles and international English articles, we conducted the Chi-Square Test, the results of which are presented in Table 2 below. The result shows that *p* = 0.038 (*p* < 0.05), revealing clear differences between the topical issues addressing in articles published in mainland Chinese and international journals. The detailed comparisons and analysis are presented in the following part in this section.

**TABLE 1:** The number and proportion of selected articles on each theme.

Theme	Mainland Chinese articles	International English articles
Measures of CSL/CFL anxiety	9 (17.31%)	11 (26.19%)
Factors associated with CSL/CFL anxiety	20 (38.46%)	10 (23.81%)
Effects of CSL/CFL anxiety	16 (30.77%)	7 (16.67%)
Classroom practices to reduce CSL/CFL anxiety	7 (13.46%)	14 (33.33%)
Total	52 (100%)	42 (100%)
		

**TABLE 2:** The chi-square test result of topical issues in mainland Chinese and international articles.

Themes	Languages (%)		Total	χ^2^	p
	Chinese	English			
Measures of CSL/CFL anxiety	9(17.31)	11(26.19)	23(24.47)	8.42	0.038*
Factors associated with CSL/CFL anxiety	20(38.46)	10(23.81)	30(31.91)		
Effects of CSL/CFL anxiety	16(30.77)	7(16.67)	20(21.28)		
Classroom practices to reduce CSL/CFL anxiety	7(13.46)	14(33.33)	21(22.34)		
Total	52	42	94		

*p < 0.05.

The measurement of anxiety level of Chinese language learners is a main topic explored by both mainland Chinese scholars and international scholars in this field. FLCAS, the most widely used tool for measuring FLA designed by Horwitz et al. (1986), was adopted in most of these studies. Apart from using FLCAS to examine learners’ listening, speaking, reading, and writing anxieties in Chinese learning (Zhang, 2002; Wu and Liu, 2019), FLCAS was also applied to test the correlation between learners’ levels of anxiety with other factors such as learners’ willingness to communicate (Liu, 2017). However, while many studies verified their research assumptions by using FLACS, Luo (2014) questioned the general reliability and validity of this anxiety scale, arguing whether the proposed FLACS could comprehensively examine students’ anxiety in Chinese language learning as it does not address the unique characteristics of the target language. In response, she developed an anxiety scale specific to the measure of CSL/CFL learners’ anxiety levels (cf., Luo, 2014).

Apart from efforts to measure CSL/CFL learners’ anxiety level, over one third of Chinese language articles and approximately one fourth of English language articles examined multiple factors associated with anxiety in CSL/CFL learning. These diverse factors can be generally categorized into two types, i.e., background variables and quantitative learner variables. Frequently inspected background variables involve learners’ gender, age of acquisition, target language proficiency, heritage-learning status, ethnic background, and the length of residence in the country where the target language is spoken. For example, Fan’s (2009) study revealed different levels of anxiety among American, Japanese, and Korean students with the same level of Chinese language proficiency when reading Chinese. This study gave further evidence that learners’ Sinosphere background, or the so-called Chinese-character cultural circle, is a significant factor that may affect language anxiety in learning Chinese. This finding is in accordance with Wen’s (2011) study published outside mainland China that compared with other ethnic groups, non-Chinese Asian groups, i.e., Asian but not Chinese American, demonstrated the most positive learning experience. In another study, Yu and Watkins (2008) found that learners’ language anxiety was significantly and negatively correlated with their Chinese language proficiency. This means that the poorer the target language proficiency, the more language anxiety they may experience. Quantitative learner variables are the second type of factors associated with anxiety of learning CSL/CFL, which include learners’ age, learning motivation, foreign language aptitude, self-perceived foreign language learning ability, language achievement, self-perceived achievement, and the difficulty level of the target language. For instance, learners’ FLA when speaking Chinese was found to be closely related to their self-perceived Chinese language proficiency in a native Chinese-speaking environment (Liu, 2017). Zhang and Wang (2002) investigated 42 international students’ FLA in learning Chinese as well as their performance in HSK (*Hanyu Shuiping Kaoshi*, standardized test of Standard Chinese language proficiency of China for non-native speakers). The analysis indicated a negative correlation between learners’ language anxiety and their Chinese language achievements, especially in listening and speaking sections. Some studies also discussed how the difficulty level of Chinese language, as an influential factor, can have an effect on learners’ anxiety. Luo’s (2015) study examined CSL/CFL anxiety and its associated factors among Chinese heritage learners. The correlation and multiple regression results indicated that perceived difficulty level of Chinese was a significant predictor of language anxiety. Overall, both mainland Chinese scholars and international scholars have made substantial contributions to exploring the various factors and their interrelationship with CSL/CFL learners’ anxiety levels.

The third main topical concern is how CSL/CFL learners’ anxiety has an impact on their language performance and achievements. As illustrated in Table 1, compared with their international counterparts, Chinese mainland researchers were more interested in this branch of research as they published almost twice as many as articles on this topic. Although findings concerning anxiety and achievement in second language learning have been relatively uniform, suggesting a consistent negative correlation between anxiety and achievement (typically test scores) (Horwitz, 2001), researchers working on anxiety of learning CSL/CFL, especially mainland Chinese scholars, have contributed to the exploration of how anxiety affects learners’ performance in specific language skills or aspects. As an example, via accurate measure of 15 international students’ voice speed, unnatural pauses during topic-oriented narration as well as levels of anxiety in learning CSL/CFL, Zhang (2001) measure the effect of anxiety on learners’ fluency of spoken Chinese. Liang and Quan (2016) examined the effect of CSL/CFL anxiety on the writing performance of ASEAN learners, i.e., students from South-East Asian Nations such as Malaysia, Thailand, and Vietnam, studying in China during different academic stages. To capture the true effects of anxiety, an English language article (Liu, 2017) assessed the effect of language anxiety together with other cultural and linguistic variables on CSL adult learners’ willingness to communicate in Chinese.

Mainland Chinese and international scholars showed different foci in the last topical concern. Although only 7 (13.46% in total) Chinese language publications explored how classroom practices could reduce CSL/CFL students’ anxiety, all of them addressed this issue from teachers’ perspectives (e.g., Deng, 2008; Guan, 2012; Tao, 2013; Liu, 2019). For example, Deng (2008) investigated overseas students’ affective reactions to teachers’ different questioning modes in CFL classrooms and concluded with suggestions for teachers to adapt their questions based on learners’ language and cognitive competence in order to bolster learners’ confidence. As another example, Tao (2013) pointed out that teachers’ inappropriate ways to correct students’ errors in oral tasks may directly lead to CFL learners’ high level of anxiety and a sense of frustration. In response, he suggested teachers decrease the frequency of correcting learners’ errors and offer them ample time to formulate their utterances. However, in these two studies mentioned above, both researchers did not further conduct comparative research to find out whether learners’ perceived anxious experience had really changed. On the contrary, among the 14 English language publications concerning this topic, most of them (10 out of 14, 71.43%) attempted to eliminate learners’ perceived anxious experience by creating learner-centered learning environment. For instance, many studies probed into the integration of VR tools (Xie et al., 2019a,b), computer-mediated communication (CMC) activities (Jiang and Ramsay, 2005; Wang et al., 2017) and on-line games (Hwang et al., 2013; Hong et al., 2019) that provide opportunities for CSL/CFL learners to immerse in the target culture and enhance their communicative competence in relaxed leaning environment. It seems that the coping strategies proposed by mainland Chinese scholars to reduce learners’ anxiety are mainly from teachers’ perspective while international scholars^3^ in this field tend to address this issue by designing learner-oriented activities or incorporating new technologies or tools to eliminate learners’ perceived anxious experience.

#### Similarities and Differences in Topical Issues

**FIGURE 3:**
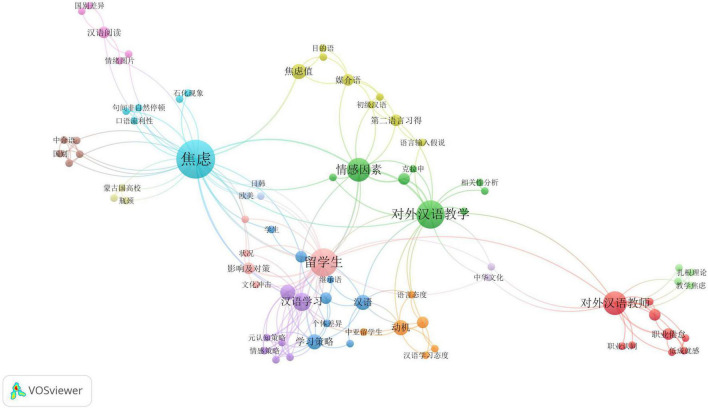
Keywords co-occurrence in Chinese language articles.

The bibliometric analysis of keywords in Chinese and English papers are presented in Figures 3, 5, respectively. In these two maps (the Chinese map and the English map), a node represents a keyword and the size of the node denotes the activity of the keyword. We set the minimum number of keyword occurrence as 1; then the threshold of the Chinese map is 250 and that of the English map is 176. In total, there are 79 items in the Chinese map and 167 items in the English map meeting the requirement. From the keyword co-occurrence network of Chinese articles shown in Figure 3, it can be seen that three keywords, i.e., “焦虑” (anxiety^4^), “对外汉语教学” (teaching CSL/CFL), and “留学生” (overseas students) play a central role and are linked to various other keywords. Closer inspection of Figure 3 reveals 14 different thematic clusters connected with each other, among which Chinese reading cluster, correlation analysis cluster, and teaching strategy cluster are the three largest clusters. It is also worth noting that the “汉语学习” (learning CSL/CFL) and 对外汉语教师 (CSL/CFL teachers) are frequently mentioned topics in Chinese language publications. Compared with the network of Chinese publications, a more complex and multi-dimensional social network structure of the English language papers is depicted in Figure 5. “Anxiety” is the biggest cluster with the highest occurrence as it was used as the search string. Although there is no definitely prominent cluster (e.g., learners/attitude/foreign language/motivation/2nd language), these 13 thematic clusters are evenly distributed, and each cluster has multiple links to each other. A comparison of Figures 3, 5 shows that “Reading” and “Speaking” are two keywords in both network structures, suggesting that issues related to speaking and reading anxieties were fully examined in Mainland Chinese and international articles. Furthermore, three keywords co-occur in these two networks of publications: “Emotion,” “Motivation,” and “Achievement,” which indicates that researchers in and outside mainland China have carried out extensive research on factors associated with learners’ anxiety in learning Chinese. This finding is consistent with our analysis in section “General observations on topical distributions of the studies.”

**FIGURE 4:**
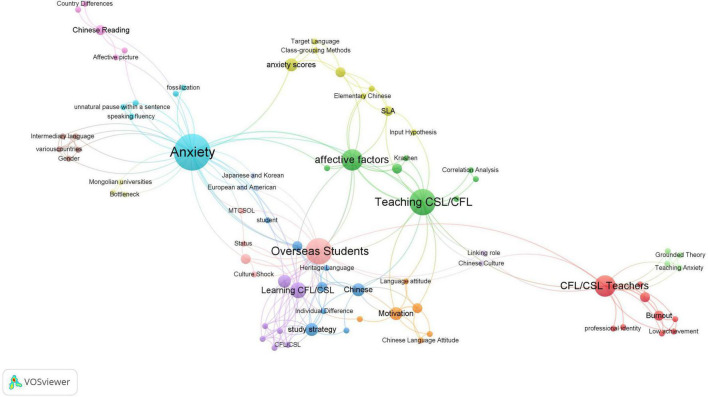
English translation of keywords co-occurrence in Chinese language articles.

**FIGURE 5:**
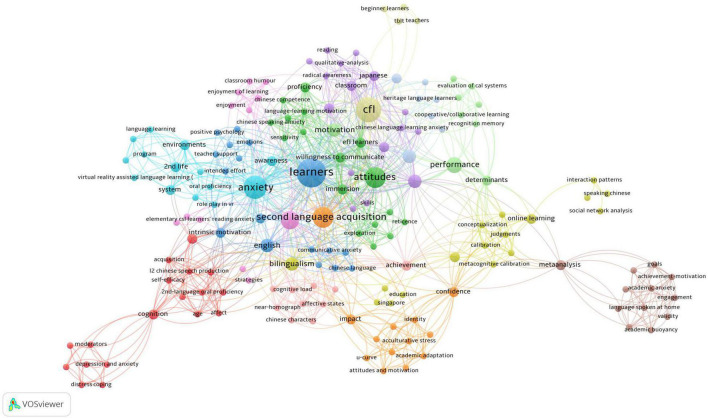
Keywords co-occurrence in English language articles.

Considering the topical distributions and the keywords co-occurrence in mainland Chinese and international journals, some differences can be identified between the research of CSL/CFL anxiety in and outside mainland China. Firstly, the comparison of the two keyword co-occurrence maps illustrates that, compared with mainland Chinese scholars, researchers overseas addressed a broader range of topical issues. In particular, some keywords such as “bilingualism,” “virtual reality assisted language learning,” “online learning” and “social network analysis” that occurred in English articles were not mentioned in their Chinese counterparts. Indeed, international researchers were aware of the importance to discuss CSL/CFL learners’ language anxiety in multilingual contexts in an attempt to explore its uniqueness in the field of SLA. As an example, McEown and Sugita-McEown (2020) assessed the effects of positive and negative psychology on students’ anxiety in learning foreign languages other than English (LOTE), i.e., Chinese, German, French, and Spanish. Their study proposed that CSL/CFL learners may experience lower levels of anxiety when compared with learners of other three LOTE in the same study environment as CSL/CFL learners generally receive more support from their parents and teachers due to the fact that Chinese (Mandarin) is the second most powerful language in the world. McEown and Sugita-McEown’s (2020) study is among a number of English language studies aimed at comparing the differences between anxiety of learning CSL/CFL and anxiety of learning other foreign languages (e.g., English) (Liu, 2016; Collie et al., 2017; Sun and Zhang, 2020), which is a new research perspective that has not been explored in Chinese language articles.

Secondly, it is noteworthy that there have been several contributions published in leading international journals addressing how the employment of information and communication technology (ICT) affect students’ anxiety in learning CSL/CFL (Wang et al., 2017; Shi and Stickler, 2018; Hong et al., 2019; Xie et al., 2019a). Wang et al. (2017), for example, found that CMC activities were an effective foreign language learning facilitator that could reduce learners’ FLA. This empirical study also indicates that CSL/CFL learners are more anxious when chatting with native speakers than chatting with non-native speakers. Shi and Stickler (2018) explored the most suitable interaction patterns between the CSL/CFL beginners and teachers of Chinese in an online language teaching environment to support online speaking practice and reduce learners’ levels of anxiety. Drawing on data obtained from the utilization of computer-assisted language learning (CALL) in CSL, Jiang and Ramsay (2005) examined whether this medium can be used to foster learner–teacher rapport beyond the realms of face-to-face interaction of the classroom. This exploratory study suggested that CALL can enhance learning, motivate learners, and reduce learner anxiety. Similarly, Xie et al. (2019b) investigated university students’ use of interactive virtual reality tools (VR tools) for learning CFL. The study indicates that the integration of VR tools in CSL/CFL classrooms not only creates an authentic language environment but also alleviates participants’ anxiety when presenting in Chinese.

In sum, by examining the commonalities and individuality of the topical issue of research on anxiety of learning CSL/CFL conducted in and outside mainland China, we can see that scholars have reached a general consensus that language anxiety is a negative emotional reaction that may interfere with CSL/CFL learning and cannot be ignored. The application of various anxiety scales, especially FLCAS in empirical studies, has greatly contributed to our understanding of levels of anxiety among a growing number of CSL/CFL learners globally. Compared with researchers in mainland China, international scholars addressed a broader range of research issues with innovative perspectives. In particular, they set research on anxiety of CSL/CFL learning in a multilingual context and make the best use of ICT in an attempt to provide coping strategies specific to CSL/CFL learners, which are rarely adopted by mainland Chinese researchers in relevant research.

### Methodological Approaches

Through examining the relevant methodological sections or descriptions in each selected article, we identified a total of 81 empirical studies and 13 non-empirical studies during the period 1999–2020 (see Table 3). The analysis illustrated that while international journals are inclined to empirical studies (100% in total), a quarter of articles published in mainland Chinese journals are non-empirical. These non-empirical studies addressed the issue of learners’ anxiety in Chinese language classrooms based on personal views without a purposed research plan, reliable data, or detailed analytical procedures (e.g., Tao, 2013; Zhao, 2013). A closer inspection of the methodological approaches adopted in the 39 Chinese empirical studies revealed that the majority of empirical studies involved quantitative research (28 out of 39, 71.79%) with only a small number of qualitative (7 out of 39, 17.94%) and mixed-method studies (4 out of 39, 10.26%). On the contrary, although quantitative research (45.24% in total) accounts for the largest proportion of English empirical studies, a large number of mixed-method studies (30.95% in total) and qualitative studies (23.81%) are also involved. It can be seemed that quantitative paradigm involving qualification of data and objective testing appealed to most mainland Chinese researchers, whereas qualitative paradigm involving naturalistic and interactive data as well as an integration of quantitative and qualitative approaches were also adopted by international researchers.

**TABLE 3:** Methodological approaches.

		Mainland Chinese articles	International English articles
Empirical studies	Quantitative studies	28 (53.85%)	19 (45.24%)
	Qualitative studies	7 (13.46%)	10 (23.81%)
	Mixed-method studies	4 (7.69%)	13 (30.95%)
Non-empirical studies		13 (25.00%)	0 (0.00%)
Total		52 (100%)	42 (100%)

A detailed examination of methods revealed that, as the most widely adopted measure for FLA, FLCAS was used in most quantitative studies published in and outside mainland China to examine language anxiety of CSL/CFL learners worldwide. In the late 1900s, Qian (1999) published the first article on anxiety of learning CSL/CFL in mainland China, using quantitative statistical methods, that is, FLCAS to measure the anxiety levels of CSL/CFL learners in target language countries. It is the first time that the concept of CSL/CFL anxiety was introduced into the research field of teaching Chinese as a foreign language (TCFL), and also the first time that the FLCAS was applied in studies of CSL/CFL anxiety. Since then, most of the relevant articles published in mainland Chinese journals used FLCAS for precise and in-depth measurement (e.g., Zhang, 2001, 2002, 2008; Zhang and Wang, 2002; Liang and Quan, 2016; Cao and Tian, 2017). A small number of studies made some adaptation to this scale for accurate measure of CSL/CFL learners’ levels of anxiety (e.g., Fan, 2009; Zhou, 2015) while most of the rest apply it directly in their studies. We speculate that this practice may result in the large proportion of quantitative studies published in mainland Chinese journals. FLCAS was also largely adopted in quantitative studies published outside mainland China. But we also notice that in order to enhance the reliability and validity of their studies, other scales were integrated with FLCAS to appropriately measure learners’ anxiety in learning Chinese as well as its correlation with other factors (e.g., Liu, 2016).

However, inspired by a number of English language studies claiming that the uniqueness of target language may have an impact on learners’ FLA (Aida, 1994; Saito et al., 1999; Le, 2004), study Luo’s (2014) published outside mainland China criticized that FLCAS, as a generic instrument, does not take the characteristics of the specific target language into consideration. In response to this lack of consideration for the target language, Luo constructed the Chinese Language Learning Anxiety Scale (CLLAS) as a reliable and valid measure for the anxiety level of CSL/CFL learning. Luo (2014) also proposed that qualitative analysis is also needed for a more comprehensive understanding of major causes of language anxiety. Consequently, qualitative techniques such as classroom discourse analysis (Lambert and Zhang, 2019), classroom observations (Xie et al., 2019b; Tong and Tsung, 2020), reflective journals (Sun and Luo, 2018), and personal interviews (Xie et al., 2019a) were used extensively in English language publications. For example, empirical work triangulating qualitative analysis of learners’ L2 discourse with multiple quantitative sources of data was conducted in order to gain deep insight into the cognitive, affective, and linguistic processes that take place on a range of pedagogical tasks in second language classrooms (Lambert and Zhang, 2019). In another study, three qualitative data collection tools, i.e., in-class observations, learners’ reflections, and semi-structured individual interviews, were carried out and aimed at probing into learners’ perceived advantages and difficulties of utilizing VR tools to learn Chinese language as well as culture (Xie et al., 2019b). Moreover, while a few Chinese language research articles (4 out of 39, 10.26%) chose a combination of the quantitative paradigm such as questionnaire surveys and anxiety scales and the qualitative paradigm including in-depth interviews and classroom observations (e.g., Deng, 2008; Cao and Tian, 2017), approximately one third of English language articles (13 out of 42, 30.95%) adopted the mixed methods approach. For instance, in a study aimed at exploring CSL learners’ anxiety when speaking Chinese and its association with self-rated proficiency in Chinese, both questionnaire surveys and semi-structured interviews were adopted (Liu, 2016). The questionnaire was designed to identify learners’ self-rated proficiency in Chinese while the interview was conducted to explore the participants’ real inner thoughts and attitudes toward learning CSL. In another study, Lambert and Zhang (2019) investigate CSL learners’ levels of anxiety in a range of pedagogical tasks by triangulating multiple sources of data including anxiety questionnaire, video-recorded classroom observation, and in-depth interviews to gain insight into learners’ cognitive and affective responses to different types of classroom tasks.

In general, while all the English language articles are empirical studies with rigorous data collection and analytical procedures, still one fourth of the Chinese language articles are non-empirical and largely based on personal experiences and reflections. In addition, most of the Chinese empirical studies report the use of statistical analysis or measurement of learners’ anxiety level. In contrast, qualitative research methods including classroom observations and personal interviews are also widely adopted in the English language articles. Moreover, mixed-method approach that entails a combination of the qualitative and quantitative paradigm with the aim of generating a more adequate and thorough understanding of CSL/CFL learners’ anxiety has been adopted largely by international researchers. This finding suggests that Chinese language research in this field has been dominated by a positivist paradigm that perceives anxiety as an objective ‘reality’ that can be scientifically studied and measured (Gao et al., 2001; Gong et al., 2018). Meanwhile, international researchers in their studies of CSL/CFL learning anxiety invested a great deal of effort in integrating theory and practice in “inquiry.” A fundamental characteristic of their research is that they are emic-oriented. That is, they attempt to interpret the complex psychological phenomenon of language anxiety from a learner’s perspective rather than from an etic or researcher’s perspective. From this perspective, qualitative research methods such as observation of authentic classroom interactions and in-depth interviews with individual participants are also essential for fully revealing the subjective and multi-faceted nature of CSL/CFL learning anxiety. Therefore, we encourage mainland Chinese scholars to be better equipped with diverse research techniques for implementing high-quality, inquiry-driven, quantitative and qualitative research. After all, methodological knowledge means not only mastery of skills, “but also informed choices and decisions” (Gao et al., 2001).

### Mainland Chinese Research’s Dependency on Previous English Language Research

**FIGURE 6:**
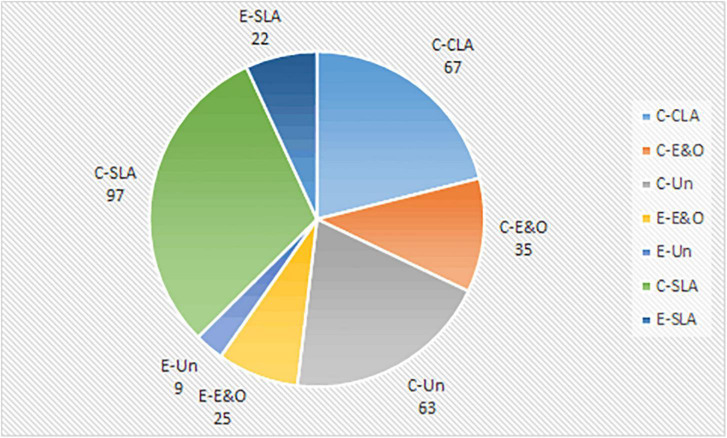
Citation trends (1999–2020). C-CLA, Chinese language publications on CSL/CFL anxiety research; C-E&O, Chinese language publications on English or other language anxiety research; C-SLA, Chinese language publications on SLA; C-Un, Chinese language publications unrelated to FLA or SLA; E-CLA, English language publications on CSL/CFL anxiety research; E-E&O, English language publications on English or other language anxiety research; E-SLA, English language publications on SLA; E-Un, English language publications unrelated to FLA or SLA.

To address our second research question, citation analysis was carried out for each selected Chinese language article by two members of the team to shed light on how these publications had drawn on relevant research published outside mainland China. In total, 318 citations were identified from the 52 Chinese language articles, including 67 Chinese language publications on CSL/CFL anxiety research, 35 Chinese language publications on English or other language anxiety research, 97 Chinese language publications on SLA, 63 Chinese language publications unrelated to FLA or SLA, 25 English language publications on English or other language anxiety research, 22 English language publications on SLA, and 9 English language publications unrelated to FLA or SLA (see Figure 6).

Overall, far more Chinese language publications (175 in total) were cited than English ones (40 in total). In addition, the analysis reveals that Chinese language articles on CSL/CFL anxiety research were cited the most (74 out of 318, 23.27% in total). This suggests that Chinese researchers draw heavily on their Chinese colleagues’ publications, especially relevant articles on CSL/CFL anxiety research. It is pretty noteworthy that no English language article on CSL/CFL anxiety research, which should belong to the most relevant and valuable intellectual base for researchers working in this field, was cited. It seems that mainland Chinese researchers do not know what their counterparts have achieved in this domain or what emerging thematic trends or new topics are being explored. This may be due to researchers’ inadequate bilingual competence (e.g., Chinese and English) and limited exchanges among researchers and institutions in China and abroad (Gong et al., 2018). Moreover, a closer examination of the 25 cited English language articles on English or other language anxiety research demonstrates that most of these articles are publications that introduced well-established research theories or concepts (e.g., Horwitz et al., 1986; Weiner, 1986; Saito et al., 1999) and very few of them are case studies that focus on a particular group of learners in specific learning contexts (e.g., Park and French, 2013). To be specific, 13 studies introduced and applied FLCAS developed by Horwitz et al. (1986) to measure CSL/CFL learners’ general level of anxiety. Furthermore, the classical “attribution theory of motivation and emotion” proposed by Weiner (1986) was introduced in Zhang (2008) and was used to verify its appropriateness to explain the causes of anxieties among foreign students learning Chinese in mainland China. Although there are a few Chinese language studies attempted to supplement and revise theories or concepts generated by international scholars, their contributions were limited to some minor revision to FLCAS in order to be used in the CSL/CFL learning context (e.g., Zhong and Gao, 2014; Wei, 2016). It seems that Chinese language scholars tend to draw on well-established and widely adopted theories generated elsewhere while failing to keep track of the latest trends in CSL/CFL anxiety research fronts.

The content analysis of the 36 cited Chinese language articles on FLA research reveals that most of these cited papers concern the identification of anxieties associated with specific skills such as reading, writing, and speaking (Chen, 1997; Kang, 2011) as well as the analysis of correlation between anxiety and affective factors (Wang and Wan, 2001; Zhou and Wang, 2008), which is in accordance with our finding of topical issues elaborated in section “Topical issues.” However, in the last decades, FLA research in mainland China, especially English language anxiety research, has witnessed an increasing number of publications adopting new theoretical perspectives and methodological approaches such as flipped teaching mode, Computer-Assisted Instruction, and online synchronous tutorials (He and Wang, 2020) which cannot be found in research on anxiety of learning CSL/CFL conducted in mainland China. A possible explanation for this finding is that most Chinese language articles on CSL/CFL anxiety are published by teachers or researchers working in CSL/CFL-related disciplines, which are sub-categories of Chines language and literature (Liu, 1997). Thereby, these researchers’ engagement with research in international contexts is not as strong as their colleagues in English-related disciplines. This can be changed if more researchers working in the field of CSL/CFL anxiety research could enhance their bilingual competence and expand their theoretical perspectives.

## Discussion

The present systematic review covers both Chinese language articles and English language articles published in flagship journals during the period from 1999 to 2020 in and outside mainland China in an attempt to depict a whole picture of the topical issues and methodological approaches and shed light on how mainland Chinese scholars have drawn on research on the anxiety of learning CSL/CFL conducted by international scholars.

Firstly, the content analysis and citation analysis of the selected Chinese publications reveal that Mainland Chinese scholars are not fully informed about the emerging theoretical trends and research fronts in terms of CSL/CFL anxiety scholarship. This is evidenced by three aspects of observations. To start with, although 42 English language publications on CSL/CFL anxiety research were identified from leading international journals, none of them was cited by their Chinese counterparts. Besides, among the 25 cited English language articles concerning English or other language anxiety, most of them are publications that introduce well-established theories or concepts published more than 20 years ago (e.g., Horwitz et al., 1986; Weiner, 1986; Saito et al., 1999). And few latest English language studies that tackle FLA from new perspectives were cited by Chinese language publications. Furthermore, a number of English language articles published in recent years that reported the incorporation of ICT into language learning in an attempt to reduce learners’ anxious and discomfort experience in classroom learning environment (Wang et al., 2017; Hong et al., 2019; Xie et al., 2019b) cannot be found in Chinese language articles in this field. The effort to apply new theoretical perspectives and methodological approach embodies international researchers’ initiative to draw on achievements of scholars from diverse disciplines to gain insight into the multi-faceted and multi-dimensional nature of FLA. Unfortunately, Chinese language researchers’ engagement with the latest research theories and concepts is relatively limited. Although Chinese scholars have drawn on relevant theories and concepts in this field, the results show that most of their research are influenced by Horwitz et al. (1986)’s FLA theory and their contributions to FLA research are rather peripheral. This failing to keep track of the emerging theoretical trends may be a result of the inadequate multilingual competence, a lack of interdisciplinary awareness, as well as the insufficient collaboration and conversations between scholars from diverse disciplines in mainland China and abroad (Gong et al., 2018).

Secondly, the content analysis reveals that research on anxiety of learning CSL/CFL conducted by mainland Chinese scholars tend to be teacher-oriented while international scholars mainly address CSL/CFL anxiety issue from learners’ perspective. Firstly, as discussed earlier in section “General observations on topical distributions of the studies,” the coping strategies formulated by mainland Chinese scholars to reduce CSL/CFL learners’ anxiety are mainly from teachers’ perspective. To be precise, mainland Chinese researchers attempted to “tell” teachers what to do, such as providing suggestions on how to group CSL/CFL learners with different nationalities (Wei, 2016), proffering advice for teachers to modify their error correction modes (Tao, 2013) or questioning modes (Deng, 2008), thereby potentially ignoring the learners’ perceived affective experience in classrooms. On the contrary, researchers outside mainland China mostly focus on learners’ language acquisition process and emotional experience during a dynamic learning process. Accordingly, they attempted to alleviate CSL/CFL learners’ perceived anxious experience by creating learner-centered learning environment, such as integrating VR tools to create authentic real-life settings for the students to feel relaxed in pedagogical practices (Xie et al., 2019b) or encouraging CSL/CFL learners to pursue and sustain interaction with faculty members, classmates as well as the host country people to better adapt to the L2 community and feel more confident and motivated in CSL/CFL learning (Yu, 2010). Secondly, the importance attached on CSL/CFL learners’ feelings and experience by international scholars can also be reflected from the methodological approaches adopted in their research. Compared with mainland Chinese articles that are largely dominated by the quantitative paradigm (28 out of 39, 71.79%), qualitative methods such as video-recorded classroom observations (Xie et al., 2019b; Tong and Tsung, 2020), semi-structured individual interviews (Xie et al., 2019a), and learners’ reflective journals (Sun and Luo, 2018) were also widely adopted in English language articles to probe into learners’ perceived anxious experience in long-term learning process. This observation is consistent with Ma et al.’s (2017) and Gong et al.’s (2018) study that Mainland Chinese researchers tend to place greater emphasis on Chinese language teachers’ beliefs, concepts, practices, and teaching pedagogy rather than learners’ values, experience, and competence. One primary reason for that may be the different educational concepts in mainland China and abroad. Chinese education emphasizes the inculcation and mastery of knowledge, emphasizing “precision” and “depth” (Li, 2007). Hence, this largely contributes to teacher-centered classrooms and teacher-oriented research perspectives. On the contrary, it has been a long tradition for international scholars especially western scholars to “focus on research into language learning and language learners/users,” harboring the fear that they might leave out learners’ voices (The Douglas Fir Group, 2016). However, to compare these different orientations, we made no attempt to build an opposition between teacher-centered and student-centered teaching practices or views of research. We would like to depict a comprehensive picture of different research perspectives so that teachers as well as scholars can make informed choices based on target students and readers.

Thirdly, our review also reveals that quantitative measure of learners’ CSL/CFL anxiety by using FLCAS (Horwitz et al., 1986) is a noticeable characteristic of most Chinese language articles. This seems to suggest that Chinese scholars studying CSL/CFL anxiety tend to view language from an “objective” perspective and expect to quantify these “objective facts,” whereas international researchers in this field are more likely to view language learning as “an activity that relied on individual psychological factors” from a more subjective perspective (Gao et al., 2001), which is evidenced by the large number of qualitative studies and mixed-method studies in the English language articles reviewed. Given that language anxiety is a subjective psychological phenomenon (Jain and Sidhu, 2013; Dewaele, 2017; MacIntyre, 2017; Boudreau et al., 2018) that may vary from person to person in different learning contexts, we encourage Chinese language researchers to be sensitive to the contextual and interactive dimensions of anxiety and adopt diverse methodological approaches. Besides, although FLCAS has contributed significantly to the study of FLA, it has received some criticism. One primary reason is that this generic instrument is designed mainly based on students’ experience of learning Indo-European languages such as English and French (Wu and Liu, 2019) and fails to address the characteristics of many other target languages such as Japanese and Chinese (Aida, 1994; Luo, 2014). Therefore, whether FLCAS remains a suitable and reliable tool to measure learners’ anxiety in CSL/CFL learning is still pending. In the light of this, Luo (2014) constructed a Chinese Language Learning Anxiety Scale in an attempt to reflect the uniqueness of the Chinese language and accurately measure CSL/CFL learning anxiety. Based on this newly developed anxiety scale, we believe that more studies to cross-validate its reliability in different learning contexts may need.

Finally, with the support of information technology, second or foreign language learning has changed from face-to-face classroom activities solely to a combination of real-life classroom interaction and virtual teaching, facilitating easy access to language learning immune to the barriers of space and time (Peters and Shi, 2011; Blake, 2013). The change of teaching platforms also entails the challenges of adapting offline pedagogical practices to online environment and tackling learners’ anxiety brought by different interaction patterns (Stickler and Shi, 2013). To reduce learners’ anxiety in synchronous online Chinese tutorials, especially during speaking practice sessions, Shi and Stickler (2018) identified the best patterns of interaction between teachers and students in an online environment that could alleviate learners’ anxiety and maximize their opportunity for speaking. This study contributes to our understanding of online teaching process and learners’ psychological state in a virtual learning environment, which is highly significant especially with the global outbreak of the COVID-19 pandemic and the urgent need to transform classroom-based teaching activities to online ones.

## Conclusion

This paper reviews the topical issues and methodological approaches of CSL/CFL anxiety research published in and outside mainland China from 1999 to 2020. The results showed that in comparison with mainland Chinese researchers, international scholars examined a wider range of topical issues in diverse contexts and dedicated to exploring the latest theoretical approach to alleviate CSL/CFL learners’ anxiety. While most Chinese empirical studies are dominated by the quantitative paradigm, qualitative and mixed-method approaches were also largely adopted by international researchers in this field. The review also reveals an evident disconnection and insufficient academic communication between mainland Chinese and international scholars in field of CSL/CFL.

To be specific, while scholars in and outside mainland China are constantly expanding their perspectives when undertaking relevant research, scholars overseas investigated a broader range of topical issues in various contexts. For example, they explored the uniqueness of CSL/CFL anxiety in a multilingual context (e.g., McEown and Sugita-McEown, 2020) and examined how learners’ anxiety in CSL/CFL learning was affected by online language education [e.g., Horwitz et al. (1986)’s FLA theory; Wang et al., 2017; Shi and Stickler, 2018]. The analysis also revealed that non-empirical studies that are largely based on personal experiences and views are absent from English language articles, whereas they account for one fourth of the Chinese language articles. In addition, most of the Chinese empirical studies are dominated by a quantitative approach that views anxiety as an objective ‘reality’ that can be scientifically studied and measured (Gao et al., 2001; Gong et al., 2018). In contrast, qualitative methods such as classroom observations and in-depth interviews are also widely adopted by international researchers to interpret the complex psycho-social phenomenon of language anxiety from a learner’s perspective. The review concludes that although Chinese language scholars have drawn on well-established and widely adopted theories and concepts originating from FLA research, they failed to keep track of the emerging trends in CSL/CFL anxiety research fronts and lacked pioneering initiative to pursue advancements in this domain. Therefore, we draw on our findings to proffer the following advice to scholars inside and outside mainland China to enhance research in this field. We hope that such efforts can help relevant research achieve greater impact in international mainstream journals.

First, it was noted in the review process that Chinese scholars working on CSL/CFL learning anxiety drawn heavily on previous literature published in mainland China and referred to limited theories and concepts appeared in international journals around two decades ago. Therefore, we strongly recommend that Chinese researchers in this field carefully review previous relevant literature published both in and outside China, so as to be well informed of the updated and emerging theoretical perspective and methodological approaches in CSL/CFL learning anxiety scholarship. For example, social network analysis (SNA) has been widely used in social and behavior science to explore relationships among diverse social entities in face-to-face encounters and the patterns formed in naturally occurring interactions (Wasserman and Faust, 1994). This method was later applied as an analytical tool in online education to reveal the best teacher–learner interaction patterns that can reduce learners’ level of anxiety during online Chinese tutorials (Shi and Stickler, 2018). We believe that this new methodological approach can offer a large amount of valuable data on diverse interpersonal relations in both face-to-face and online language classrooms, the result of which has implications for teachers to enhance their competence to modify interaction patterns and create low-anxiety classroom atmosphere. Besides, previous research on the integration of CALL in language learning has proffered solid evidence of how CALL, compared with face-to-face classroom interaction, can promote interactive learning (Warschauer, 2000), “genuine communication” (Bax, 2003, p. 23) in target languages, build up teacher–learner rapport and alleviate learners’ anxiety (Jiang and Ramsay, 2005). However, how and to what extent can CALL foster the social relationship between teacher and students in CSL/CFL teaching context remain a neglected area of research and need more future studies. At last, apart from the frequently inspected variables that are associated with CSL/CFL learning anxiety such as learners’ ethnic background (Fan, 2009) and Chinese language proficiency (Yu and Watkins, 2008), Yu (2010) observed that international students’ sociocultural and academic adaptation also closely related to their language learning attitudes and level of anxiety. This finding has implications for both researchers and teachers to lay more emphasis on how CSL/CFL students from abroad integrate into local communities and host institutions. The introduction of new methodological approaches and theoretical perspectives to CSL/CFL anxiety research may facilitate scholars to cast new light on this complex psychological phenomenon and enable classroom practitioners to alleviate CSL/CFL learners’ feeling of nervousness and apprehension.

Second, the content analysis as well as the bibliometric analysis of the selected Chinese language publications reveals a tendency to address the issue of CSL/CFL learners’ anxiety mainly from teachers’ perspective. Chinese scholars may now want to take diverse perspectives and gain insight into this topical concern from learners’ perspective. This means that apart from giving instructions to teachers on how to implement some specific teaching practice or strategy and view learners as passive beneficiaries of teachers’ conduct (e.g., Deng, 2008; Tao, 2013; Wei, 2016), language researchers as well as classroom practitioners can design student-oriented activities or learner-generated content tasks (e.g., Lambert and Zhang, 2019) in which learners can gain relaxed and enjoyable experience directly. The integration of virtual reality tools into CFL learning in an attempt to create an authentic setting and alleviate learners’ anxiety when delivering presentations in a foreign language is a good example to follow (e.g., Xie et al., 2019b). Furthermore, starting from learners’ point of view also requires the researchers to view CSL/CFL learning anxiety as a subtle, fluid and subjective psychological phenomenon. This means that the “pure” quantitative methods such as the use of questionnaires (e.g., Hoxur, 2001) or anxiety test scale (e.g., Zhang, 2002) only may not be adequate to accurately measure learners’ anxiety during a dynamic learning process. Driven by the specific research questions, qualitative methods such as classroom observations, writing reflective journals and individual interviews that can generate comprehensive and naturalistic data may also needed. This should encourage researchers to equip themselves with qualitative techniques as well as rigorous data collection and analytical and interpretation methods in order to conduct high-quality research.

Last but not least, we hope that more researchers could draw their attention to how and to what extent CSL/CFL learners’ anxiety levels are affected by online or remote language teaching, which becomes even more inevitable and ubiquitous with the advent of the COVID-19 pandemic. On the one hand, it has been documented that computer-mediated communication has been a popular method in foreign language teaching (Chun et al., 2016) due to the fact that it can increase motivation toward foreign language learning (Abrams, 2011) and facilitate a more comfortable and relaxed learning environment (Doughty and Long, 2003; Jochum, 2011). On the other hand, the sudden change of classroom settings, the lack of direct interaction between teachers and learners, the weak emotional bond, and the unstable internet access are all potential variables that can affect foreign language learners’ level of anxiety (Mahyoob, 2020). Therefore, we urge scholars in and outside mainland China to work collaboratively to address the critical issues related to anxiety of CSL/CFL learners globally emerging from the virtual language education environment. Moreover, opportunities and resources in and outside CSL/CFL classrooms need to be integrated to improve learners’ Chinese proficiency and communicative competence in daily situations, which can be a crucial and useful means to reducing their learning anxiety (Gong et al., 2020b, 2021).

The current study has several limitations. First, given that the journal publications are valued more than other types of publications in institutional research assessment exercises, only the journal articles published in the CSSCI and SSCI journals were reviewed, whereas the non-CSSCI/SSCI journal articles, books, or book chapters were excluded, which might devalue the investigation on the account of leaving out some valuable academic monographs. Second, in view of the sociocultural and historical differences between diverse learning contexts, the articles published from Taiwan, Hong Kong, and Macau were also excluded. Therefore, various types of publications (i.e., books) and database sources (i.e., Taiwan Social Science Citation Index) can be included in the future studies to provide more insights into CSL/CFL anxiety research.

## Data Availability Statement

The original contributions presented in the study are included in the article/supplementary material, further inquiries can be directed to the corresponding author/s.

## Author Contributions

SY, DZ, and QS contributed to conception and design of the study. SY organized the database. DZ performed the statistical analysis. QS wrote the first draft of the manuscript. All the authors contributed to manuscript revision, read, and approved the submitted version.

## Conflict of Interest

The authors declare that the research was conducted in the absence of any commercial or financial relationships that could be construed as a potential conflict of interest.

## Publisher’s Note

All claims expressed in this article are solely those of the authors and do not necessarily represent those of their affiliated organizations, or those of the publisher, the editors and the reviewers. Any product that may be evaluated in this article, or claim that may be made by its manufacturer, is not guaranteed or endorsed by the publisher.

## Footnotes

1 The term “international journals” used in this article refers to journals issued outside mainland China and the term “international articles” refers to articles published in international journals.

2 The group 3 refers to publications concerning issues of second language acquisition that are not associated with foreign language anxiety. As the full explanation would be too long, we use “Chinese language publications on second language acquisition” to name this group. This also applies to group 7.

3 As English is now the most powerful global language (Ma et al., 2017) and the main medium of academic publication (Gong et al., 2020a), the term “international scholars/researchers” is used in this article to refer to scholars/researchers who tend to publish their articles written in English outside mainland China.

4 The English translation of each Chinese keyword can be found in Figure 4.
